# Physiological effects of human body imaging with 300 mT/m gradients

**DOI:** 10.1002/mrm.29118

**Published:** 2021-12-21

**Authors:** Malwina Molendowska, Fabrizio Fasano, Umesh Rudrapatna, Ralph Kimmlingen, Derek K. Jones, Slawomir Kusmia, Chantal M. W. Tax, C. John Evans

**Affiliations:** 1 Cardiff University Brain Research Imaging Centre (CUBRIC), School of Psychology, Cardiff University, Cardiff, United Kingdom; 2 Siemens Healthcare Ltd, Camberley, United Kingdom; 3 Siemens Healthcare Gmbh, Erlangen, Germany; 4 Faculty of Health Sciences, Mary McKillop Institute For Health Research, Australian Catholic University, Melbourne, Australia; 5 Image Sciences Institute, University Medical Center Utrecht Imaging Division, Utrecht, The Netherlands

**Keywords:** gradient switching fields, magnetic fields, magnetophosphenes, peripheral nerve stimulation, ultra-strong gradient system

## Abstract

**Purpose:**

The use of high-performance gradient systems (i.e., high gradient strength and/or high slew rate) for human MRI is limited by physiological effects (including the elicitation of magnetophosphenes and peripheral nerve stimulation (PNS)). These effects, in turn, depend on the interaction between time-varying magnetic fields and the body, and thus on the participant’s position with respect to the scanner’s isocenter. This study investigated the occurrence of magnetophosphenes and PNS when scanning participants on a high-gradient (300 mT/m) system, for different gradient amplitudes, ramp times, and participant positions.

**Methods:**

Using a whole-body 300 mT/m gradient MRI system, a cohort of participants was scanned with the head, heart, and prostate at magnet isocenter and a train of trapezoidal bipolar gradient pulses, with ramp times from 0.88 to 4.20 ms and gradient amplitudes from 60 to 300 mT/m. Reports of magnetophosphenes and incidental reports of PNS were obtained. A questionnaire was used to record any additional subjective effects.

**Results:**

Magnetophosphenes were strongly dependent on participant position in the scanner. 87% of participants reported the effect with the heart at isocenter, 33% with the head at isocenter, and only 7% with the prostate at isocenter. PNS was most widely reported by participants for the vertical gradient axis (67% of participants), and was the dominant physiological effect for ramp times below 2 ms.

**Conclusion:**

This study evaluates the probability of eliciting magnetophosphenes during whole-body imaging using an ultra-strong gradient MRI system. It provides empirical guidance on the use of high-performance gradient systems for whole-body human MRI.

## 1 Introduction

Technical advances in gradient performance (including the availability of higher amplitude gradients) have led to tremendous improvements in MR imaging. In recent years, various high-performance head-only^1–6^ and whole body^7,8^ gradient systems have been developed, which confer performance benefits for microstructural imaging^9–11^ in research and clinical studies.^12–16^ However, rapid switching of ultra-strong gradient systems also produces rapidly time-varying, strong, magnetic fields which have physiological effects on the human body. These phenomena effectively limit the extent to which ultra-strong gradients technologies can be used safely for in vivo imaging.

The interaction of electrical fields with nerves and muscles was established by George Weiss,^17^ and nature of this interaction was widely investigated by Eccles, Hodgkin and Huxley.^18–22^ Nerve stimulation occurs when the application of an extra-axonal electric field pulse, above a certain threshold and parallel to the main axis of the axon, generates an action potential. Reilly^23^ described the phenomenon of peripheral nerve stimulation (PNS), an involuntary muscle twitch, induced by rapidly time-varying magnetic fields produced by switching gradient coils on and off. MR systems equipped with gradients stronger than 100 mT/m can produce other physiological effects,^7^ including magnetophosphenes^24^ or, in extreme cases, respiratory^25^ or cardiac muscle stimulation, for example, an ectopic heartbeat.^26,27^ Magnetophosphenes are most likely generated by stimulation of the retina. They are generally perceived as a sensation of flashing or a faint flicker spanning much of the field of view, but often reported in peripheral vision. Magnetophosphenes are a biologically reversible effect and thus are considered non-harmful. They are, however, a sensitive probe to the lower thresholds of the human physiological response to time-varying magnetic fields and thus, they are considered useful in establishing thresholds of effects which are biologically irreversible.^28^

Electrophysiological models, based on the spatially extended nonlinear node (SENN) model,^21^ indicate that the applied electrical field must exceed a given threshold to generate an action potential. This threshold depends on the characteristics of the extracellular stimulus, for example, single pulse or multiple pulses, monophasic or biphasic pulses, and the delay between consecutive pulses.^23^ Efforts have been made to design gradient pulse shapes that minimize PNS effects, especially for MRI acquisitions such as echo-planar imaging (EPI).^29–31^.

As defined by the IEC Standard ISO/IEC-60601-2-33,^32^ the PNS limits for a scanner can be established by studying the PNS reported by a cohort of volunteers. During routine scanning, a dedicated model, implemented in system hardware and software, predicts the specific physiological limits for a given measurement.^33^

The Siemens 3T Connectom is whole-body system with ultra-strong gradients (300 mT/m gradient amplitude and 200 T/m/s slew rate).^7,8,11^ The system was originally designed for neuroscience research but there is increasing interest in using the system’s ultra-strong gradients “below the neck”, for example, advanced diffusion applications in the heart and prostate.^34^

The production of magnetophosphenes had not previously been a problem with maximal achievable gradient amplitudes (e.g., ≤80 mT/m). However, initial work on an ultra-strong gradient system^7^ reported magnetophosphenes when using gradient strengths greater than 130 mT/m, but only when the eyes were located more than 10 cm away from the isocenter. Thus, this physiological effect is particularly relevant for body applications, when the eyes are far away from the isocenter.

The IEC Standard ISO/IEC-60601-2-33^32^ does not define a regulatory limit for magnetophosphene stimulation, nor a procedure for defining this limit in practice. The aim of this study was to evaluate the probability of eliciting magnetophosphenes to develop a practical guideline for minimizing participant discomfort and/or anxiety.

## 2 Methods

### 2.1 Participants

Ethical approval for the study was obtained from the School of Psychology Research Ethics Committee of Cardiff University. Fifteen participants were recruited for this study (age range: 27–50, M = 38.87, *SD* = 7.17, weight: M = 77.73, *SD* = 9.59 kg, height: M = 178.6, *SD* = 6.39 cm; M, mean; SD, standard deviation). All participants were male (to define a prostate landmark) and had no disclosed medical problems that would influence the validity of the results. Participants had provided informed consent prior to participation and had taken part in previous MRI studies. Participants were informed in advance that the aim of the study was to investigate physiological effects and were briefed on the likely physiological effects that might be experienced to ensure that these effects could be detected.

### 2.2 Experimental setup

**Figure 1:**
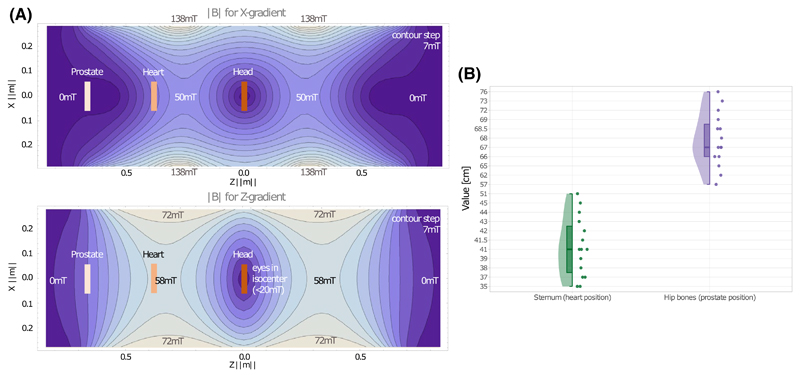
A, Contour plots of the absolute magnetic field generated by the gradient coil used in this study. *X*-axis corresponds to left-right, *Y*-axis to anterior–posterior, and *Z*-axis to head–foot directions. To aid in the interpretation of the magnetophosphene results, the approximate position of the eyes for the prostate, heart, and head positions are shown (rectangular boxes). *Top row:* |*B*| for a 300 mT/m *X*-gradient (*Y* = 0). Note the plot for the *Y*-gradient is substantially equivalent for the illustrative purposes of the current figure. *Bottom row:* |*B*| for a 300 mT/m *Z*-gradient (*Y* = 0). B, Descriptive analysis of measured absolute distances between the nasal area and the body landmarks for the study population. Dots represent measured lengths, the half violin plot presents the distribution of measurements in the study sample and the half box plot represent the median and interquartile range: 41 cm, IQR = 5 cm and 67 cm, IQR = 2.75 cm for heart and prostate landmarks, respectively. Please note that *Y*-axis grid spacing is not uniform

The imaging system used in the study was a Connectom MRI scanner, a modified 3T MAGNETOM Skyra system fitted with an AS302 gradient coil capable of 300 mT/m (Siemens Healthcare, Erlangen, Germany). To assist in providing a reference to other works, the vendor supplied maps of the maximum absolute magnetic field of the gradient coil (excluding main magnet **B**_0_) allowing the peak d***B***/d*t* during the experiment to be estimated (Figure 1A).

Participants were placed on the scanner table in the supine position prior to data collection from three anatomical regions: head (head first), heart (head first), and prostate (feet first). In each case, the participants’ left arm was positioned flat on the table alongside the left hip and their right hand on an optical fibre-interfaced 5 button response box (LxPad, NATA Technologies, Coquitlam, Canada) placed on the abdomen. This device was used to receive feedback from the participants on the experience of the physiological effects. In line with our institute’s standard procedures, the participants were also supplied with an alarm call button and a pulse oximeter (placed on the left hand index finger) to monitor the cardiac cycle. The participants were instructed not to move. The scanner vendor’s standard pneumatic headphones and earplugs were used to limit acoustic noise. To increase the sensitivity to any visual effects, the ambient light in the scanner room was reduced to a minimum; the magnet and control room lights were switched off and any remaining light was from monitor displays. Lastly, absolute distances between the nasal area and the end of the sternum (heart position) and hip bones (prostate position) were measured (Figure 1B).

### 2.3 Acquisition protocol

We assessed the occurrence of magnetophosphenes in participants when applying a continuous train of 128 trapezoidal bipolar gradient pulses, similar to that used in a conventional EPI read-out. Each gradient pulse train was applied along a single gradient axis with ramp times ranging from 0.88 to 4.20 ms and gradient amplitudes from 60 to 300 mT/m. These ranges were constrained by the MR system’s physiological limit monitors. The gradient amplitude/ramp time combinations used in the study were defined by performing a preliminary investigation of magnetophosphenes on three MR-experienced participants.

Data were sampled from the lowest gradient/shortest ramp time to the strongest gradient/longest ramp time (70 combinations, gradient variation first), for each of the three imaging landmarks (head, heart, and prostate), for each gradient axis (*X*, *Y*, and *Z*), resulting in 630 stimuli for each participant. The sampling of the gradient/ramp time combinations was weighted towards the high-gradient/long-ramp time measurements, as the low-gradient/short-ramp time measurements had been previously performed by the vendor.

The gradient axes are defined as follows: When standing in front of the scanner and looking into the magnet, the *X*-axis points from left to right (horizontal axis); *Y*-axis points from bottom to top (vertical axis); and *Z*-axis points from rear to front (depth axis). After each gradient stimulus, the participants were asked to indicate via a button press whether they experienced; (a) no effects, (b) PNS, and/or (c) magnetophosphenes. In the event of any other perceived effects, participants were instructed to use the scanner alarm call button to report this to the experimenters. The participants were not aware of the order of applied gradient amplitude/ramp time stimuli. The only imaging performed was a localizer for landmark identification using the whole-body RF coil to verify participant positioning.

From the observations across the cohort, an estimate of the probability of encountering physiological effects was established; the probability was calculated as a ratio of counts of the reported effect (PNS or magnetophosphenes) to the number of samples collected for each ramp time and gradient amplitude combination.

A post-scan questionnaire was administered immediately after the experiment. The questionnaire was designed to capture any other subjective effects not reported by the participants during the tests.

## 3 Results

All study participants successfully completed the experiment and were able to provide valid responses during the experiment and feedback via the post-scan questionnaire.

**Figure 2:**
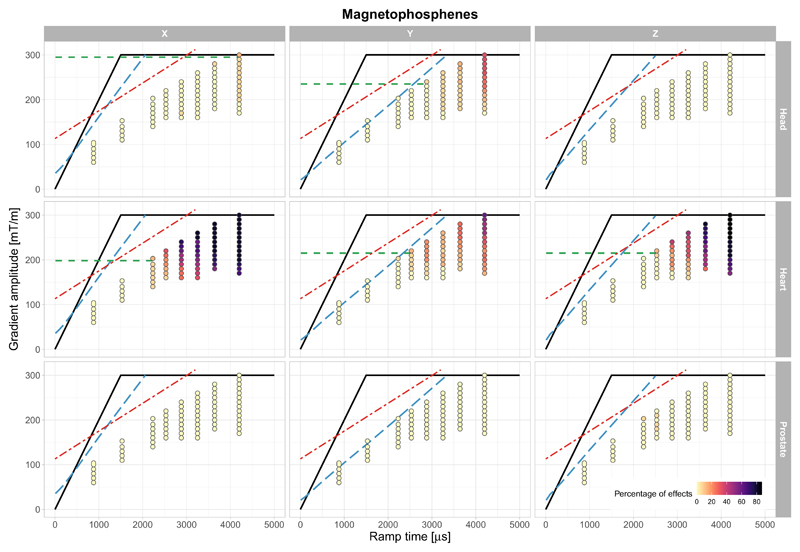
Percentage of participants reporting magnetophosphenes for the three imaging landmarks and three gradient axis. The dots are color-coded according to the percentage of volunteers reporting the effect for each of ramp time/gradient amplitude pair. The hardware limit (maximum slew rate and maximum gradient amplitude) is depicted as solid black line curve, the approximate location of the cardiac stimulation limit is shown as red dot-dashed line and the PNS limit (SAFE model,^33^ for *X*, *Y*, and *Z* axes separately) is shown as blue dashed line. As outlined in the discussion, the magnetophosphene guideline value, *G_amp_*, is shown as a green dashed line. This represents the maximum gradient amplitude, at the maximum slew rate, for which fewer than 10% of participants reported magnetophosphenes. This is not defined where the guideline value is greater than 300 mT/m

Magetophosphenes were reported by most participants in at least one landmark position, as shown in Figure 2. These visual effects were more widely reported when the gradient amplitude was above 150 mT/m, where the gradient ramp times are longer. For the highest gradient amplitudes, when the corresponding gradient stimulus ramp times were above 3 ms, magnetophosphenes were reported by up to 86.7% of the participants (for the heart landmark); for lower gradient amplitudes, when ramp times were below 3 ms, fewer than 30% of participants reported magnetophosphenes.

Participant reports of magnetophosphenes varied greatly between experiments performed in the different landmark positions. For the heart landmark, 80%, 60 %, and 86.7% of the study participants reported perceivable changes in vision during the experiment, for the *X*, *Y*, and *Z*-axes, respectively. In the other landmark positions, there were fewer reports of magnetophosphenes, notably in the prostate position (6.7% of participants). For the head position, 33.3% participants reported magnetophosphenes when the stimulus was applied along the *Y*-axis; however, these were rarely reported on the *X*-axis (13.3%) and not reported at all when pulsing the *Z*-axis.

**Figure 3:**
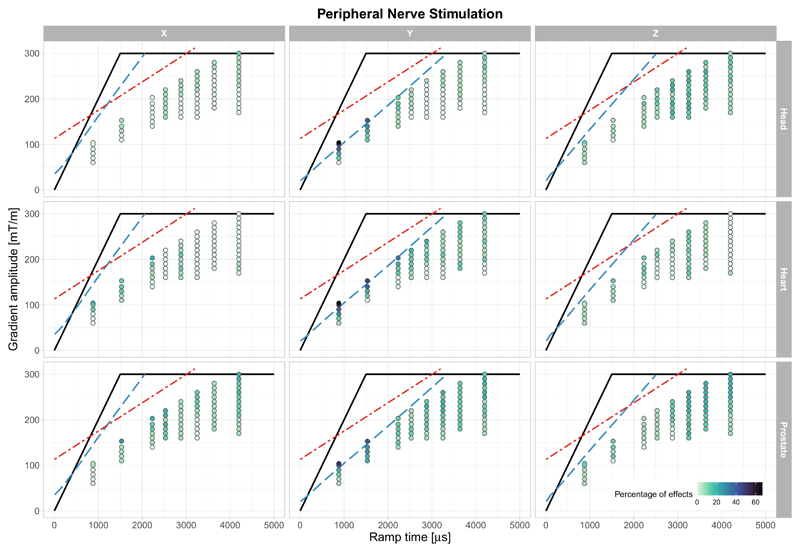
Percentage of participants reporting PNS for the three imaging landmarks and three gradient axes. The dots are color-coded according to the percentage of volunteers reporting the effect for each of ramp time/gradient amplitude pair. The hardware limit (maximum slew rate and maximum gradient amplitude) is depicted as solid black line curve, the approximate location of the cardiac stimulation limit is shown as red dot-dashed line and the PNS limit (SAFE model,^33^ for *X*, *Y*, and *Z* axes separately) is shown as blue dashed line

The highest incidence of PNS (Figure 3 and Table 1) occurred when the gradient was applied along the *Y*-axis, where 66.7% of the study volunteers reported some degree of PNS (head or heart positions). In contrast to magnetophosphenes, PNS was less common when applying gradients with longer ramp times, with participants reporting PNS in less than 20% of the cases when the ramp time was greater than 3 ms. PNS was less prominent when the stimulus was applied along the *X* or *Z*-axis. There was no dramatic effect of imaging landmark on the PNS probability, as can be seen from Table 1.

The participants reported PNS across a wide range of locations in the body: in the shoulders, lower back, neck, jaw and below the ears, chest, abdomen, arms, fingers, legs, and feet. There was no consistent spatial pattern of PNS occurrences.

**Figure 4:**
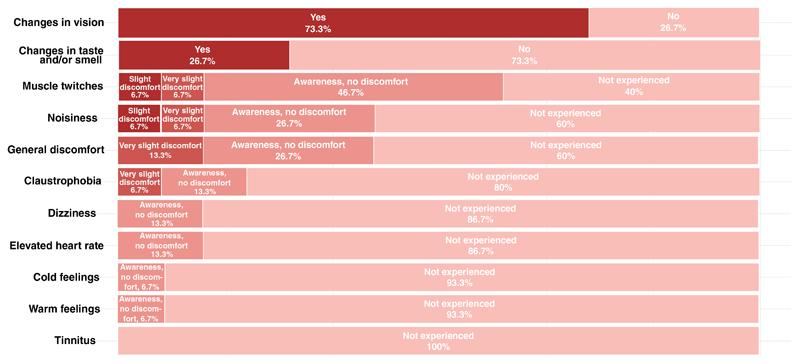
The post-scan questionnaire results presenting the frequency of other physiological effects experienced during the scan

The post-scan questionnaire responses are reported in Figure 4. Overall, 13.3% of the participants reported some level of discomfort during the experiment. This discomfort was associated with muscle twitches (13.3%), claustrophobia (6.7%), or scanner noise (13.3%). In all cases, these were classified by participants as either a “very slight discomfort” or a “slight discomfort”.

The most commonly reported effects were changes in vision by 73.3% of the participants, and muscle twitches which were reported by 60% (although primarily classified as an “awareness of the effect,” rather than discomfort). Other effects reported by participants were changes in smell or taste, dizziness, elevated heart rate (participant’s subjective sensation, not evidence from pulse-oximeter measurements) and changes in temperature. In each case, these were reported as an “awareness” rather than “discomfort”. These effects were already reported in the literature, and are most likely not related to gradient switching.^10^

## 4 Discussion

We established the magnetophosphene stimulation probability in three anatomical locations, for a trapezoidal train of gradient pulses up to 300 mT/m. The experiment was implemented within limits imposed by regulatory bodies on PNS and cardiac stimulation.

While there have been several reports of magnetophosphenes associated with MRI, most are associated with the movement of an individual within the main (static) ***B***_0_ field.^35,36^ To our knowledge, only two previous studies have reported the observation of phosphenes in participants arising from gradient switching, one in a similar system^7^ and the other from a dedicated head only gradient coil.^37^ There is renewed interest in application-specific gradient coils, for example for neuroimaging applications.^38^ These gradient coils offer the performance advantages of higher slew-rates and greater gradient strength, while their reduced gradient fringe fields offer improvements in PNS performance when compared with whole-body gradient coils.

Electrophysiological studies have demonstrated that the magnetic field threshold for producing magnetophosphenes depends on the magnetic field switching rate with the minimum threshold occurring at around 30 Hz.^39^ The gradient switching frequencies used in this study are significantly higher, ranging between approximately 60 Hz and 300 Hz. However, the magnetophosphene occurrence reported in this work is consistent with earlier work, showing a reduction in the magnetophosphene threshold with a reduction in the switching frequency (i.e., longer ramp times). This effect was observed for all axes along which magnetophosphenes were observed.

At the location of the eyes, the estimated maximum absolute magnetic field due to the gradient coil alone, at maximum output (300 mT/m, Figure 1) is ~20 mT, ~50 mT and ~20 mT for the head, heart, and prostate landmarks, respectively. The magnetophosphenes reported by participants during the study (Figure 2) are consistent with these data—lower gradient amplitudes produce magnetophosphenes when the heart is at isocenter, than when the head or prostate are at isocenter. As a comparison with existing literature, Lovsund^39^ reported thresholds of ~10 mT for a stimulus of 30 Hz and ~14 mT at 45 Hz. In our results, magnetophosphenes were consistently observed for gradient amplitude 150 mT/m and ramp time 4.2 ms in the head position (*X*-gradient) equivalent to **B**_max_ of approximately 25 mT at ~60 Hz.

No participants suffered discomfort due to the experience of magnetophosphenes, and they are not believed to be associated with any long-term health effects.^28^ Phosphene generation by magnetic, electrical and/or mechanical stimulation of retina and cortex are well known and generally reversible (non-harmful) phenomena.^40–42^ For example, visual phosphenes are commonly experienced in brain stimulation techniques and these phosphenes have been attributed to a combination of cortical and retinal effects.^43–45^

Due to the variability in participants’ physiological responses, participant positioning, gradient waveforms, and MR hardware, it is challenging to condense these results into general guidance for the treatment of magnetophosphenes in MRI research. We have attempted to do so, acknowledging a number of caveats, by using the following approach: For each condition (gradient axis and landmark), a “magnetophosphene guideline value" was defined, representing the maximum gradient amplitude (*G*_amp_), at the maximum slew rate, for which fewer than 10% of participants reported magnetophosphenes. These guideline values are shown in Figure 2, for each gradient axis and landmark. Please refer to the Supporting Information section for a detailed description of the guideline definition (Supporting Information Figure S1).

Studies exceeding the maximum *G*_amp_ should take additional measures before routinely scanning research participants. Such additional measures will depend on the institution’s local rules but should include steps such as participant informed consent, Institutional Review Board approval, monitoring and/or limiting the duration of the exposure, pilot studies to evaluate stimulation in novel sequences/protocols/waveforms and additional monitoring of the participant during scanning (e.g., MR safe pulse oximeter or interaction via the intercom). Ideally, pilot studies should include the determination of probability maps, such as the ones determined in this work, to take advantage of the full potential of high-performance gradient systems.

The reports of PNS presented here are also in line with previous findings,^46,47^ namely that the PNS probability is higher along when pulsing the *Y*-gradient, most likely due to the larger anatomical cross-sectional area normal to the *Y*-axis. At ramp times longer than 1.5 ms, fewer reports of PNS were obtained (33.3% of participants across all imaging landmarks and gradient axis). A fraction of these PNS reports may have been sensations of gradient coil vibrations which are substantial at higher gradient amplitudes. When participants were uncertain, these observations were included as a report of PNS. Although the intensity of the PNS was reported to increase with higher amplitudes of the applied impulse, comparison to literature is not feasible; in the previous works, protocols were stopped upon reaching the physiological threshold.^38,48^

We questioned participants on any aspects of discomfort during the study to investigate for any unexpected effects of high gradient field systems.^10^ The reported effects, such as elevated heart rate, dizziness or a sensation of warming during the scan, were consistent with reports from participants in studies on other systems in our center. However, in our study, changes in vision (related to gradient switching, not static magnetic field) were more consistently reported, especially for high gradient amplitudes, as expected due to change of body position within scanner bore.

### 4.1 Study limitations

The sampling of the gradient/ramp time points was limited by the scanner: the PNS limit (as implemented by the SAFE model^33^) and the IEC cardiac limiter^32^ (as implemented via a hardware limit on gradient output) were both enabled during the study. Additionally the set of gradient/ramp time measurement points was kept consistent for all gradient axes. This results in the *X* and *Z* gradients reporting a lower PNS probability than the *Y*-axis. As a result, it was not possible to define a physiological threshold in all cases, for example, the phosphene threshold usually exceeds the existing scanner’s cardiac limit for ramp times shorter than 3 ms. This is due to a conservative interpretation of the IEC cardiac limit, based on Reilly extrapolation from animal studies,^27^ which was set to an estimated probability of 10^−9^ to produce an ectopic beat (ISO/IEC-60601-2-33).

The magnetophosphene guidelines yield indicative values for when further investigation is required when planning a study involving a novel gradient waveform or pulse sequence. Further work will be conducted to investigate physiological effects in applications using alternative diffusion encodings such as multidimensional q-space imaging,^49^ and to evaluate the consistency of the magnetophosphene guidelines on the application of different waveforms. For generalized gradient encoding, as well as for newly developed gradient systems,^4,37^ further work is required to predict the stimulation patterns precisely.^48^ Comparisons with other studies using bipolar gradient schemes can be impaired by slight differences in the timing between the studies.^38,48^

As our main interest was in the longer ramp time/stronger gradient domain, a limited dataset was acquired in the gradient/ramp time combinations more commonly sampled during PNS studies (less than 100 mT/m and 1 ms). As a consequence, the conclusions that can be drawn relating to PNS are limited.

Finally, the participant group contained only males (due to the interest in the prostate). Thus the magnetophosphene and PNS probabilities may be different for females, adolescents or younger groups, as differences in anatomical distances will lead to participants experiencing different parts of the gradient field (Figure 1).

## 5 Conclusions

Evaluation of the physiological limits is critical for human safety on high-performance gradient MR systems. This study investigated the elicitation of magnetophosphenes by a high-gradient field system when different anatomical locations were placed at isocenter. Based on the framework provided by this work, the likelihood of magnetophosphenes can be estimated in future studies of different anatomical regions when using ultra-strong gradient whole-body MR systems.

## Supplementary Material

Supplementary Material

## Figures and Tables

**Table 1:** Summary of the perceived physiological effects (magnetophosphenes and PNS) in three body imaging landmarks for the *X*, *Y*, and *Z* axes

	Magnetophosphenes			PNS		
	*X*	*Y*	*Z*	*X*	*Y*	*Z*
Head	13.3%	33.3%	0.0%	13.3%	66.7%	20.0%
Heart	80.0%	60.0%	86.7%	20.0%	66.7%	13.3%
Prostate	0.0%	0.0%	6.7%	26.7%	53.3%	20.0%

*Note*: Values represent the highest percentages of participants experiencing effects for each landmark-axis pair among the tested ramp times-gradient amplitude combinations.
