# Supplementary material for: Physiological effects of human body imaging with 300 mT/m gradients
Source: Magn Reson Med. Author manuscript; Available in PMC 2023 Oct 26. (PMC7615249; doi:10.1002/mrm.29118)
Supplement: Supplementary Material [file EMS189816-supplement-Supplementary_Material.pdf]

Supporting Information for manuscript "Physiological Effects of Human Body Imaging with 300 mT/m Gradients"

Malwina Molendowska[1], Fabrizio Fasano[2,3], Umesh Rudrapatna[1], Ralph Kimmlingen[3], Derek K. Jones[1,4], Slawomir Kusmia[1], Chantal M.W. Tax[1,5], C. John Evans[1,*]

1. Cardiff University Brain Research Imaging Centre (CUBRIC), Cardiff University, Cardiff, United Kingdom
2. Siemens Healthcare Ltd, Camberly, United Kingdom
3. Siemens Healthcare GmbH, Erlangen, Germany
4. Centre for Medical Image Computing, University College London, London, United Kingdom
5. Image Sciences Institute, University Medical Center Utrecht, Utrecht, The Netherlands

* Corresponding author:

**Name**	C. John Evans
**Department**	School of Psychology
**Institute**	Cardiff Universtiy Brain Research Imaging Centre (CUBRIC), Cardiff University
**Address**	Maindy Road<br>CF24 4HQ<br>United Kingdom
**E-mail**	evansj31@cardiff.ac.uk

Definition of the practical guideline for magnetophosphenes

We evaluated the potential of magnetophosphenes for limiting the peak performance of an experiment by defining a 'magnetophosphene guideline value' which can be used to evaluate the probability of magnetophosphenes in a new experiment. For each condition (gradient axis and landmark), a 'magnetophosphene guideline value' was defined, representing the maximum Gradient amplitude (G_{amp}), at the maximum slew rate, for which fewer than 10% of participants reported magnetophosphenes (Figure 2, in the main manuscript).

An example for Y axis - heart landmark condition is shown in the Figure S1 below; all maximum gradient, maximum slew rate points are indicated by arrows, with green arrows representing the points where less than 10% of participants reported magnetophosphenes and red arrows for those points where more than 10% of participants reported the effect. The shaded area represent all points for which more than 10% of participants reported magnetophosphenes. In the example below, the guideline value is defined by the point at 203 mT/m at a rise time of 2230 ms; resulting in a guideline value of 203 mT/m for studies acquiring data with high gradient strengths on the Y axis with the participant positioned for cardiac imaging.

In some situations, the gradient/rise time combinations which would be required to accurately define the threshold (moderate gradient strength $\sim$ 50-150 mT/m, but very long ramp times $>$ 2ms) are not practically useful due to their inefficiency from a sequence design perspective. As a result these points were not included in the study and thus, this guideline excludes any information on low gradient amplitude-long risetime combinations. The proposed guideline holds for the case where the gradient is being ramped near the maximum slew rate.

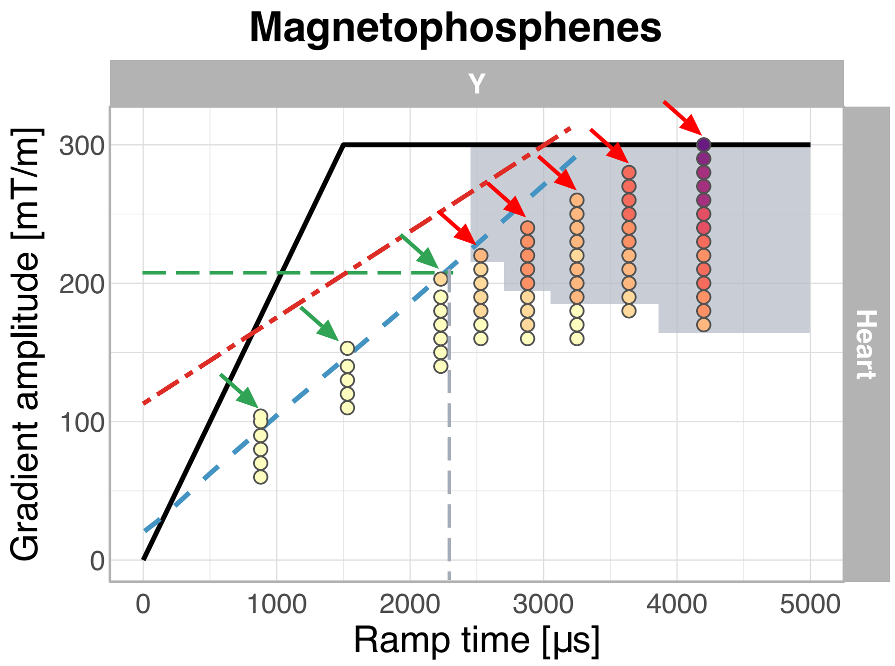

Figure S1: Visualisation of 'magnetophosphene guideline value' definition.
